# Methodological and regulatory considerations for causal AI in drug development

**DOI:** 10.1038/s41746-026-02477-w

**Published:** 2026-02-27

**Authors:** Hana Lee, Sky Qiu, Spencer Haupert, Gabriel K. Innes, Tristan Naumann, Demissie Alemayehu, Mark van der Laan

**Affiliations:** 1 Center for Drug Evaluation and Research, U.S. Food and Drug Administration (FDA), Silver Spring, MD USA; 2 Division of Biostatistics, School of Public Health, University of California, Berkeley, CA USA; 3 Microsoft Research, Redmond, WA USA; 4 Pfizer, Inc., New York, NY USA

**Keywords:** Computational biology and bioinformatics, Drug discovery, Mathematics and computing, Medical research, Scientific community

## Abstract

Advances in AI offer significant opportunities to enhance drug development. While several regulatory agencies have begun issuing guidance on AI adoption, its application to causal inference—a critical piece to understand treatment effects and inform regulatory decisions—remains limited. This paper reviews regulatory activities and examines statistical methodologies for AI-driven causal inference. We discuss key regulatory challenges and illustrate how AI adds value across diverse data sources and studies.

## Introduction

The rapid advancement of artificial intelligence (AI), including machine learning (ML), technologies has ushered in a new era of possibilities in drug development and evaluation. Since 2016, the United States (US) Food and Drug Administration (FDA) Center for Drug Evaluation and Research (CDER), which regulates medical drug products, has received more than 800 submissions with AI components^1^. In recognition of the growing interest and potential use of AI in drug development, the FDA and other regulatory agencies have instituted measures to foster the proper implementation of these technologies across the entire continuum of a drug product life cycle. Over the last several years, the FDA CDER has published several key documents and convened workshops on the use of AI in medical product development, with the goals of providing a clear regulatory framework, emphasizing the responsible use, and promoting external stakeholder engagement. The key publications from the FDA include:

- The 2021 Good Machine Learning Practices for Medical Device Development document^2^, a joint work by the US FDA, Health Canada, and the United Kingdom’s Medicines and Healthcare products Regulatory Agency. This document provides 10 guiding principles to promote safe and effective AI-enabled medical devices, which are also applicable to drugs and biologics development.
- A discussion paper titled “*Using Artificial Intelligence & Machine Learning in the Development of Drug & Biological Products*” that outlines opportunities and challenges associated with AI use across the drug development life cycle^3^.
- A document titled “*Artificial Intelligence & Medical Products: How CBER, CDER, CDRH, and OCP are Working Together*” that highlights cross-center collaboration and shared regulatory priorities on AI^4^.
- A draft guidance titled “*Considerations for the Use of Artificial Intelligence to Support Regulatory Decision-Making for Drug and Biological Products* “ that introduces a 7-step risk-based credibility assessment framework for evaluating AI models based on their context of use^5^.

Other global regulatory efforts include the European Medicines Agency (EMA)’s reflection papers on AI in the medicinal product lifecycle, which advocates for a human-centered, risk-based approach^6,7^. The International Council for Harmonization of Technical Requirements for Pharmaceuticals for Human Use (ICH) also published the M15 guideline on general principles for model-informed drug development^8^.

A central theme in these AI guidance is a risk-based assessment — evaluating model influence and consequence of wrong decision (risk). To ensure the responsible use of AI, regulatory agencies emphasize data quality and integrity (including bias mitigation), model transparency, explainability and interpretability, the necessity of human oversight, and multidisciplinary collaborations. The FDA’s AI guidance additionally stresses the importance of providing detailed pre-specification in regulatory submissions.

To our knowledge, no regulatory agency has developed guidance specifically addressing AI for treatment effect estimation and causal inference, although several FDA documents briefly cover the potential of AI for closely related areas such as analysis of electronic health records (EHRs) and medical claims data^9^. Traditionally, AI/ML have been primarily deployed for prediction tasks such as classification and forecasting, prioritizing predictive accuracy over mechanistic understanding. Causal inference, however, seeks to establish a cause-and-effect relationship, focusing on contrasting outcomes in the presence and absence of a treatment condition. In this context, key objectives include not only the accurate estimation of treatment effects but also the rigorous quantification of uncertainty, i.e., optimizing the bias-variance trade-off. Given the growing, proactive regulatory activities on the appropriate use of AI in drug development, and the central role of causal inference in understanding treatment effects and guiding decisions about drug efficacy and safety, it is timely to examine how AI can be applied responsibly in this context.

In the following sections, we review statistical methodologies that support the development of an AI for causal inference, namely, Causal AI. We highlight key challenges in using these methods for regulatory purposes, provide practical insights into responsible and effective application, and address common misconceptions. Finally, we discuss emerging opportunities for applying the causal AI for drugs and biologics product development and evaluation.

## AI for causal inference

Below, we provide an overview of statistical methods that can leverage AI for causal inference, outline key challenges and considerations for their use in regulatory settings, and highlight opportunities for broader application. This review focuses on methods for confounding control. Approaches such as instrumental variables, colliders, mediation, and methods that do not involve inference (e.g., constructing causal graphs) are beyond the scope.

### Overview of statistical methods for causal AI

Propensity score (PS)-based methods are commonly used in practice for causal inference, where the PS is defined as the probability of receiving treatment given covariates. Examples of PS-based methods include PS matching and inverse probability of treatment weighting^10,11^, for which various matching and weighting methods are available^12–17^. The PS can also be used for stratification or regression adjustment^18,19^.

PS is a balancing covariate, meaning that two or more groups with the same (or similar) PS values are balanced with respect to covariates used to estimate the PS. Therefore, PS-based methods typically rely on correct specification of the PS model particularly when parametric modeling of PS is considered. Because the selection of covariates and their functional forms in the PS model are important, various ML algorithms can be used to estimate PS more flexibly, reducing sensitivity to model misspecification. A wide range of ML-based methods have been proposed and performance assessment using simulation/empirical studies has been conducted^20–26^.

G-computation is a maximum likelihood-based approach that models the outcome as a function of treatment and confounders and standardizes over the empirical covariate distribution to estimate counterfactual mean outcomes^27^. G-computation relies on correct specification of the outcome model. One can use parametric regressions or ML for outcome modeling, with ML-based methods offering greater flexibility and reduced sensitivity to model misspecification. All ML methods for modeling PS can also be applied to modeling outcome.

Doubly robust (DR) estimators combine the treatment modeling (i.e., PS) with the outcome regression, providing unbiased estimates if either model is correctly specified, thereby offering two chances to get the answer right^28–30^. With the use of ML, DR methods can be even less sensitive to the correct model specification assumptions.

It is crucial to note that the naïve application of ML for modeling the PS and/or outcome does not guarantee valid inference. Coyle and van der Laan (2018) demonstrated that establishing the statistical property necessary for valid inference in large samples is challenging in this setting^31^. This is because ML prioritizes prediction accuracy of PS and/or outcome response, which is distinct from the ultimate goal of treatment effect estimation and inference. To properly leverage ML for causal inference within a DR framework, double or debiased machine learning^32^ or targeted maximum likelihood estimation (TMLE)^33–35^ should be considered.

Various Bayesian methods have also been proposed for the purpose of PS estimation^36,37^, G-computation^38–40^, and DR estimation^41,42^. In addition, the integration of AI with Bayesian network-based approaches which provides a synthesis of causal effects (with uncertainty quantification) by aggregating diverse clinical evidence has been discussed^43,44^. In this framework, AI can be leveraged for various tasks, such as screening studies for relevance via text mining and learning key parameters (e.g., Bayes factors) from historical data.

Table 1 provides a summary of these methods, including their key strengths and limitations, and considerations for pre-specification for their regulatory use.

**Table 1:** Summary of causal inference methods

Method	Brief Description	Key Strengths	Key Limitations	Pre-specification for regulatory use
PS methods	Model the probability of treatment assignment to create comparable groups (i.e., emulating a randomized clinical trial). Forms the basis of PS matching and weighting.	Straightforward design; clear separation of design and analysis; directly mimics a randomized clinical trial; easy to communicate across disciplines.	Sensitive to PS model misspecification. Loss of power with matching; increased variance and poor performance under limited covariate overlap with weighting. Naïve application of ML to estimate the PS may lead to invalid inference.^a^	A complete set of confounders or a detailed, reproducible, outcome-blinded process for confounder identification. Parametric models for PS and/or outcome regression, or a pre-specified, reproducible data-adaptive approach. If a data-adaptive approach is used, a valid variance estimation procedure (e.g., influence function-based approach or targeted bootstrap^a^) must be pre-specified.
G-computation	Model the outcome to estimate potential outcomes under different treatment conditions. Compare average predicted (potential) outcomes across treatment conditions to estimate the target (treatment) effect.	Uses all available data; extendable to complex settings, including time-varying treatments.	Relies on correct specification of the outcome model. Naïve application of ML for outcome modeling may lead to invalid inference.^a^	
DR methods	Model both the PS and the outcome. If at least one of the models is correct, the estimated target effect is deemed correct.	More robust to model misspecification than PS or G-computation method.	Requires fitting two models, increasing complexity; performance may degrade if both models are misspecified. Naïve application of ML for modeling the PS and outcome may lead to invalid inference.^a^	
TMLE	A DR method with an additional targeting step designed to reduce bias and enable valid statistical inference.	Typically outperforms standard DR estimators. Can incorporate ML or Super Learner (SL) while ensuring valid inference. When both the PS and outcomes are correctly estimated, result in the smallest variance (i.e., the most efficient method)	Conceptually and computationally complex. May offer limited interpretability for understanding treatment assignment mechanisms and their relative contributions.	A complete set of confounders. Outcome-blinded, automated confounder screening may be pre-specified as part of the SL library. See Gruber et al. (2023)^b^ for additional key pre-specification considerations.
Bayesian	Can be integrated with PS methods, G-computation, and DR estimators. Applicable to evidence synthesis across studies.	Allows incorporation of prior information. Provide direct probability of the causal effect (e.g., 92% posterior probability that treatment A reduces the risk of the outcome compared with control.)	Specification and justification of priors can be challenging. Conceptually and computationally complex.	A complete set of confounders or a detailed, reproducible, outcome-blinded process for confounder identification. Detailed information on external data sources used to inform priors, including borrowing strategies and assessment of study comparability.

*PS* propensity score, *DR* doubly robust, *TMLE* Targeted maximum likelihood estimation, *ML* machine learning, *SL* Super Learner.

^a^Coyle, J., & van der Laan, M. J. (2018). Targeted bootstrap. In Targeted Learning in Data Science: Causal Inference for Complex Longitudinal Studies (pp. 523–539). Cham: Springer International Publishing.

^b^Gruber, S., Lee, H., Phillips, R., Ho, M., & van der Laan, M. (2023). Developing a targeted learning-based statistical analysis plan. Statistics in Biopharmaceutical Research, 15(3), 468–475.

### Challenges and considerations

The use of AI inevitably presents several challenges including overfitting, a lack of transparency around underlying assumptions, and difficulties with interpretability^45,46^. We discuss key challenges and considerations specifically related to their use for causal inference in regulatory settings, and explore potential solutions.

The first challenge is selecting the best-performing algorithm for a given dataset, particularly with the rapid pace of advancement and wide range of available AI methods. No single algorithm is universally optimal, and it is generally difficult to determine a priori which algorithm will perform best for a specific task. To address this, Super Learner (SL) has been proposed as a principled, data-driven ensemble method that leverages multiple algorithms^47^. SL uses an objective performance criterion called cross-validation to evaluate and combine predictions from a user-specified library of candidate algorithms, selecting either the optimal weighted combination of the algorithms or a single algorithm with the best performance. A key theoretical property of the SL is that when sample size is large, it performs as well as the best algorithm in the library^48,49^. By enabling diverse algorithms to compete and guiding the best selection, SL provides a robust, flexible framework for achieving strong predictive performance across a wide range of datasets and tasks.

While SL helps mitigate bias from model misspecification, the next challenge in building sound causal AI is ensuring valid statistical inference. As mentioned in Section “Overview of Statistical Methods for Causal AI”, directly applying AI methods for prediction tasks (even with SL) to estimate the PS and/or the outcome model often results in biased effect estimates and unreliable variance, even in large samples^31^. This leads to inflated Type I error rate (i.e., incorrectly concluding a treatment is effective) and poor confidence interval coverage (i.e., failing to capture the true effect 95% of the time), a significant concern from a regulatory perspective. One potential solution is to integrate AI within the context of TMLE, which utilizes AI specifically for causal inference tasks by optimizing the bias-variance trade-off, rather than minimizing prediction error.

The final challenge centers on ensuring transparency, with an emphasis on the pre-specification of the entire analysis plan. FDA’s draft guidance on AI describes a process for establishing the credibility of AI model outputs and describes the key information that should be included in a sponsor’s proposal for each model^5^. For example, the draft guidance recommends providing descriptions of model inputs and outputs, model features, feature selection processes, model parameters, and the data used for model development. Additionally, we’d like to emphasize that pre-specification of the following information is important when AI is considered for the analysis of primary and/or secondary outcomes in a regulatory submission: performance metric, number of folds for cross-validation, candidate learners in the library and their specifications if SL or any ensemble method is considered, fixed random seeds, software versions, data preprocessing steps, and details of the computational environment. As an illustrative case, Gruber et al. (2023) demonstrate how TMLE with SL can be fully pre-specified for causal inference, offering a practical example of how to achieve both transparency and reproducibility of AI for causal inference^50^.

### Opportunities across study designs, data sources, and disease areas

As briefly noted in Section “Overview of Statistical Methods for Causal AI”, most casual AI methods have been developed in the context of confounding control in observational studies. This reflects the origins of many AI techniques, which are often designed to handle large-scale, complex, and high-dimensional datasets. In this regard, one particularly promising area for AI is the analysis of real-world data (RWD)—including EHRs, medical claims, and other sources—for generating clinical evidence of a treatment effect, i.e., real-world evidence (RWE).

RWD often contains a large (and sometimes extremely large) number of covariates, many of which are likely to be highly correlated. This high dimensionality makes the pre-specification of covariates for modeling the treatment mechanism and outcome regression particularly challenging. Moreover, traditional parametric modeling assumptions (e.g., main-term regression models for outcome analysis) are often overly restrictive or impractical. AI methods offer a promising solution by enabling data-driven covariate selection and flexible modeling strategies that reduce reliance on strong parametric assumptions, thereby improving robustness and credibility of RWE.

The FDA’s discussion paper already include discussions about the potential of AI in combination with RWD/RWE for a range of applications (yet, outside the context of causal inference) including matching patients to trials, analysis of RWD, postmarketing surveillance, continuous monitoring of AI models, identification of previously unrecognized drug effects, endpoint development, digital twins, and outcome assessment^3^. It briefly mentions TMLE for treatment effect estimation, though without detailed considerations for its use. More experience and application are likely needed to support the use of AI for analyzing RWD to generate reliable RWE.

**Fig. 1:**
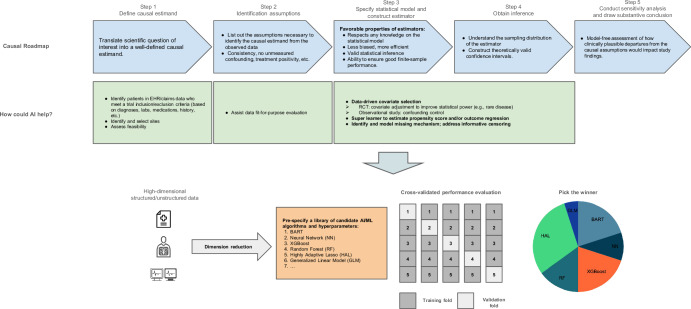
A general causal inference workflow and potential areas for AI integration.

We emphasize that generating high-quality RWE relies on a rigorous scientific workflow, as stressed by Hernan, Wang, and Leaf^51^ and Dang et al.^52^, among others. The study should start from a clear definition of the treatment effect of interest (referred to as the *estimand* in statistical literature)^53^; the data should be fit for regulatory purpose^9,54^; and study design including analysis method should be adequate^55^. In addition, the underlying assumptions of the method along with plausible deviations and their impact for robustness of findings should be carefully planned before study initiation^53^. Figure 1 outlines a general causal inference workflow and identifies potential areas for AI integration at each stage.

Although the origins of AI may be rooted in applications to high-dimensional observational data, it is important to recognize that the potential of causal AI extends beyond this context. At least two common misconceptions warrant clarification. First, AI is sometimes viewed as useful only for analyzing observational data and not for RCTs. Second, AI methods are often assumed to be applicable only to large, complex data, but not to small sample data.

Regarding the first misconception, it is important to note that in RCTs, treatment assignment is independent of covariates by design. In other words, concerns about treatment model misspecification are minimal because any covariate effects in the treatment model should be negligible. This ensures that when DR-based AI is applied to RCT data, the resulting treatment effect estimates remain unbiased while potentially more efficient. Moreover, statistical theory demonstrates that using estimated PS, rather than known true values, can improve statistical efficiency. Additionally, in longitudinal RCTs where informative censoring may occur, modeling the censoring mechanism with ML or SL can further reduce bias.

Addressing the second perception, AI methods have great potential for analysis of rare diseases (i.e., those affecting approximately 200,000 individuals in the US or fewer than 50 patients worldwide^56^). These settings often present unique challenges, including highly limited sample size, poorly characterized natural history, heterogeneity in disease presentation, and gaps in scientific knowledge on prognostic factors of outcomes. As a result, analysis of rare disease RCTs often suffers from restricted statistical power to detect the true effect. While covariate adjustment can help increase statistical power, expert guidance on which variables and outcomes are most relevant is often lacking, making it challenging to identify and pre-specify relevant prognostic factors and prioritize outcomes for statistical analysis. In addition, model misspecification (particularly, missing important prognostic factors in analysis model) may result in a substantial loss of statistical power. In such settings, leveraging AI that can systematically identify and adjust for data-driven prognostic factors while being less sensitive to the model misspecification may be highly valuable, regardless of the study design (RCTs vs. others). Lastly, in the presence of informative censoring, methods that can further reduce bias can be particularly beneficial by reducing bias while maintaining efficiency.

## Discussion and concluding remark

We have reviewed key regulatory activities, particularly those by the FDA, regarding the use of AI in drugs and biologics development. As highlighted in this manuscript, there is growing recognition and evolving guidance around the responsible use of AI for medical product development. In light of expanding discussions in this field, the central role of causal inference, and the rapid methodological advances in causal AI with its potential to transform drugs and biologics development, our goal has been to provide an in-depth assessment of the associated challenges, key considerations, and emerging opportunities for regulatory science.

Although this manuscript has focused on the use of AI for drugs and biologics development processes, the FDA’s discussion paper outlines the use of AI across the entire product lifecycle, including postmarket activities and manufacturing settings. Causal AI can be particularly useful for postmarket safety studies, an area where the FDA has actively explored and gained extensive experience^57,58^. The FDA’s Sentinel System for medical product safety surveillance, which utilizes EHRs and claims data for more than 100 million people from participating healthcare systems, has expanded its analytic capacity to incorporate AI-enabled causal inference methods^59,60^. The intersection between causal AI and RWD for pharmacovigilance & pharmacoepidemiology is also an active area of research as well^61,62^. Finally, advanced analytics leveraging AI in pharmaceutical manufacturing could improve various aspects of the supply chain—for example, enhancing demand forecasting, production schedule, and inventory optimization—thereby supporting more reliable healthcare delivery and promoting public health.

To fully harness the value of AI for treatment effect estimation and inference, sustained collaboration among regulators, industry, and academia will be essential. With careful pre-specification, rigorous planning, and oversight, AI-driven methods can play a transformative role in how new therapies are developed, evaluated, and ultimately delivered to patients.

## Data Availability

No datasets were generated or analysed during the current study.
